# Endovascular Treatment of a Direct Carotid Cavernous Sinus Fistula with the Woven EndoBridge Aneurysm Embolization System

**DOI:** 10.1007/s00062-020-00914-1

**Published:** 2020-05-30

**Authors:** Andrei Tropine, Abdul Moussa-Pacha, Stephanie Tritt

**Affiliations:** Institute for diagnostic and interventional Radiology and Neuroradiology, Helios HSK Wiesbaden, Wiesbaden, Germany

## Introduction

A carotid-cavernous fistula (CCF) is a rare type of acquired arteriovenous shunt between the internal carotid artery (ICA) and the cavernous sinus (CS). The CCFs are commonly classified into four types (A–D) according to Barrow [1]. The type A CCF is characterized by a direct high-flow shunt between the ICA and CS. This type of fistula is usually caused by trauma or rupture of a cavernous aneurysm [1]. Caused by the arterialization of the superior ophthalmic vein and venous congestion, clinical presentations of direct CCF include acute proptosis, chemosis, headache, and visual impairment due to nerve palsy [1–4]. The most common treatment of choice is endovascular transvenous embolization with coils or embolic material [5]. This article presents a case of direct CCF caused by a ruptured cavernous aneurysm treated with a Woven EndoBridge (WEB) device (MicroVention, Tustin, CA, USA). To our knowledge, this is the first time such an approach has been reported.

## Case Presentation

**Fig. 1:**
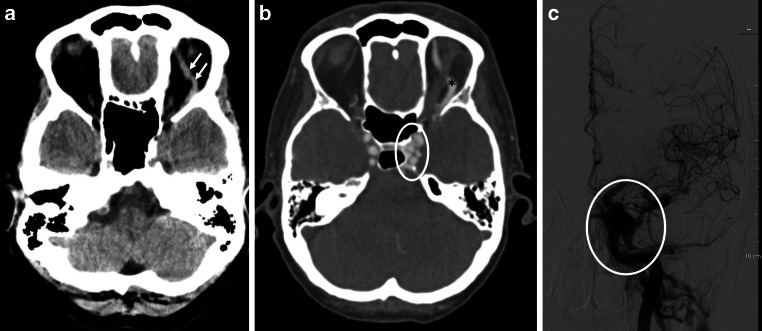
**a**, **b** Initial CCT showed a dilated ophthalmic vein and arterialization of the CF (*arrows*, *asterisk*, *ellipse*); DSA **c** revealed a high-flow direct CCF

**Fig. 2:**
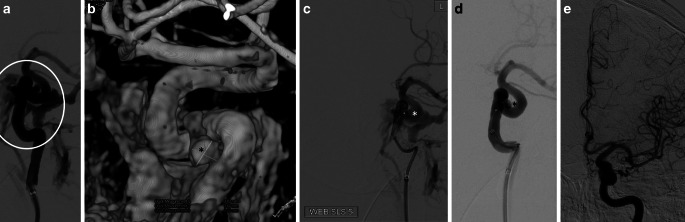
DSA (**a**) and 3D reconstruction detected a ruptured aneurysm on the ICA, which was the reason for the direct CCF (**b** *asterisk*). A Single-Layer-Spheric (SLS) WEB device was positioned in the aneurysm (**c** and **d**). The control series after showed complete occlusion of the direct CCF (**e**)

This article reports about a 54-year-old female patient who presented with acute ophthalmological symptoms with exophthalmos, chemosis with subconjunctival hemorrhage and pulsatile tinnitus. A history of trauma was not reported. Emergency cranial computed tomography (CCT) with CT angiography showed arterialization of the left CS as well as massive dilatation of the left superior ophthalmic vein (Fig. 1a, b). The patient was transferred to the angio suite and digital subtraction angiography (DSA) was performed with the patient under general anesthesia. The angiography revealed the presence of a direct fistula from the left ICA to the left CS at the level of the C4 segment (Fig. 1c). No participation of branches of the external carotid artery was visible. The reconstruction of the 3D rotational angiography showed an irregularity of the cavernous carotid artery which was suggestive of a ruptured aneurysm at this level of the vessel wall (Fig. 2a, b). The suspected aneurysm was approximately 3 × 4 mm in size in the 3D measurements.

## Treatment

**Fig. 3:**
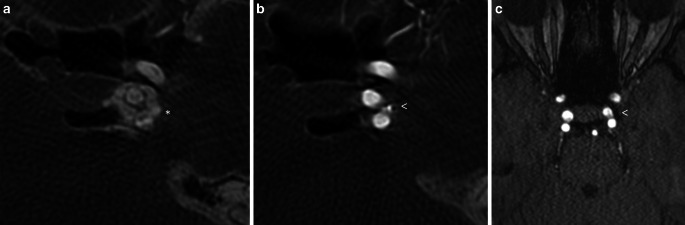
Axial multiplanar reformation of 3‑D rotational angiography showed the fistula point before (**a**) and after (**b**) implantation of WEB device (*arrow* shows a proximal marker of the WEB). Follow-up MR angiography (TOF in axial view) showed complete occlusion of the fistula and correct positioning of the WEB (**c**)

The cavernous segment of the ICA was carefully passaged by a microwire to place the tip of the microcatheter into the suspected aneurysm. According to the suspected size of the aneurysm, a SLS 5 WEB was deployed with the proximal marker landing only just in the lumen of ICA. The device was deployed in a cork-like configuration. The control series before the detachment of the device already showed a significant reduction in the arterial flow in the left cavernous sinus (Fig. 2c, d). The following control series after about 10 min showed complete occlusion of the fistula and the device was successfully detached without secondary dislocation or movement. The final examination showed a normalized flow in the internal carotid artery and the cavernous sinus (Fig. 3c). We purposely refrained from giving an antithrombotic medication in order to achieve the desired effect of intra-device thrombus formation to occlude the CCF. We do not take into account the amount of heparin, given in the permanent flushing.

## Outcome and Follow-up

The following days the patient showed a marked improvement in clinical symptoms with complete resolution of the chemosis and visual impairment. Follow-up control after 6 months, both clinical and magnetic resonance imaging (MRI) and DSA, showed stable results. At long-term follow-up 12 months later, there was no recurrence of clinical symptoms or any evidence for fistula recurrence in MR angiography.

## Discussion

Various methods have been described for the treatment of direct CCFs, including either the use of coils, volume or hydrogel coils, or a liquid embolization as well as flow diverters (FD), such as single or combined methods [6]. The development of the various methods for treating CCF underlines the progress of modern interventional neuroradiology. So far, the use of intra-aneurysmal flow disruptor like the Woven EndoBridge (WEB) aneurysm embolization system has not been described in the treatment of CCF. The WEB, as self-expanding intrasaccular device with dense microwoven mesh structure, leads to a disruption of the intra-aneurysmal flow and following to intra-aneurysmal and intra-device thrombus formation [8]. This effect could be explained by the changes in intra-aneurysmal blood flow, probably mainly by decreasing of outflow velocity than inflow velocity [9]. The safety and technical efficacy of the WEB in the treatment of ruptured and unruptured aneurysms have been described in many studies and it is nowadays a well-established treatment option [7]. The treatment of a direct CCF caused by a ruptured aneurysm of the ICA has not been described in the current literature. This case report showed that this approach might be a safe and technical feasible treatment option in Barrow A CCF. In order to verify this method, further studies should be carried out, especially in cases involving larger fistula openings and high shunt volumes. One limitation could be that in the cases with a high-flow shunt due to the wide opening of the fistula (e.g. in large aneurysms or traumatic CCF) it might be not possible to occlude the fistula but only by the use of the WEB device. In these patients, both transvenous coiling as a well-established option and FD as a new option can be used.

## Conclusion

This report of a single case in the use of the WEB device shows an alternative possibility in the treatment of a direct CCF and feasibility of this new method.

## References

[1] Barrow DL, Spector RH, Braun IF, Landman JA, Tindall SC, Tindall GT (1985). Classification and treatment of spontaneous carotid-cavernous sinus fistulas. J Neurosurg.

[2] Han W, Kim JH, Kang HI, Kim DR, Moon BG, Kim JS (2019). Transvenous embolization of dural carotid cavernous fistula through the supraorbital vein. J Cerebrovasc Endovasc Neurosurg.

[3] D’Angelo L, Paglia F, Caporlingua A, Sampirisi L, Guidetti G, Santoro A (2019). Atypical manifestation of direct low-flow carotid-cavernous fistula: Case report and review of the literature. World Neurosurg.

[4] Ertl L, Brückmann H, Patzig M, Fesl G (2019). Endovascular therapy of direct dural carotid cavernous fistulas—A therapy assessment study including long-term follow-up patient interviews. PLoS ONE.

[5] Barranco Pons R, Da Ros V, Scaggiante J, Muniz da Silva G, Picchi E, Di Giuliano F, Aja Rodriguez L, Chirife Chaparro O (2019). Transradial comaneci-assisted coiling of a direct carotid-cavernous fistula. Radiol Case Rep.

[6] Zanaty M, Chalouhi N, Tjoumakaris SI, Hasan D, Rosenwasser RH, Jabbour P (2014). Endovascular treatment of carotid-cavernous fistulas. Neurosurg Clin N Am.

[7] Arthur AS, Molyneux A, Coon AL, Saatci I, Szikora I, Baltacioglu F, Sultan A, Hoit D, Delgado Almandoz JE, Elijovich L, Cekirge S, Byrne JV, Fiorella D (2019). The safety and effectiveness of the Woven EndoBridge (WEB) system for the treatment of wide-necked bifurcation aneurysms: final 12-month results of the pivotal WEB Intrasaccular Therapy (WEB-IT) Study. J Neurointerv Surg.

[8] Lescher S, du Mesnil de Rochemont R, Berkefeld J (2016). Woven Endobridge (WEB) device for endovascular treatment of complex unruptured aneurysms—A single center experience. Neuroradiology.

[9] Gölitz P, Leucking H, Hoelter P, Knossalla F, Doerfler A (2020). What is the hemodynamic effect of the Woven EndoBridge? An in vivo quantification using time-density curve analysis. Neuroradiology.

